# Music training enhances the automatic neural processing of foreign speech sounds

**DOI:** 10.1038/s41598-017-12575-1

**Published:** 2017-10-03

**Authors:** Bastien Intartaglia, Travis White-Schwoch, Nina Kraus, Daniele Schön

**Affiliations:** 1 Aix Marseille Univ, Inserm, INS, Inst Neurosci Syst, Marseille, France; 2 Auditory Neuroscience Laboratory and Department of Communication Sciences, Northwestern University, Evanston, Illinois United States of America; 3 Department of Neurobiology, Northwestern University, Evanston, Illinois United States of America; 4 Department of Otolaryngology, Northwestern University, Chicago, Illinois United States of America

## Abstract

Growing evidence shows that music and language experience affect the neural processing of speech sounds throughout the auditory system. Recent work mainly focused on the benefits induced by musical practice on the processing of native language or tonal foreign language, which rely on pitch processing. The aim of the present study was to take this research a step further by investigating the effect of music training on processing English sounds by foreign listeners. We recorded subcortical electrophysiological responses to an English syllable in three groups of participants: native speakers, non-native nonmusicians, and non-native musicians. Native speakers had enhanced neural processing of the formant frequencies of speech, compared to non-native nonmusicians, suggesting that automatic encoding of these relevant speech cues are sensitive to language experience. Most strikingly, in non-native musicians, neural responses to the formant frequencies did not differ from those of native speakers, suggesting that musical training may compensate for the lack of language experience by strengthening the neural encoding of important acoustic information. Language and music experience seem to induce a selective sensory gain along acoustic dimensions that are functionally-relevant—here, formant frequencies that are crucial for phoneme discrimination.

## Introduction

Music and language are universals of human culture, and both require the perception, manipulation, and production of complex sound sequences. These sequences are hierarchically organized (syllables, words, sentences in speech and notes, beats and phrases in music) and their decoding requires an efficient representation of rapidly evolving sound cues, selection of relevant information, construction of temporary structures taking into account syntactic rules, and many other cognitive functions. It is thus not surprising that music and speech processing share common neural resources^1–4^, although some resources may be distinct^5^. The acoustic and structural similarities as well as the shared neural networks between speech and music suggest that cognitive and perceptual abilities transfer from one domain to the other via the reorganization of common neural circuits^2^. This hypothesis has been verified by showing that musical practice not only improves music sound processing^6–9^, but also enhances several levels of speech processing, including the perception of prosody^10^, consonant contrasts^11^, speech segmentation^12^ and syntactic processing^13^. Interestingly, these findings extend to the subcortical level, showing an enhancement of the neural representations of the pitch, timbre, and timing of speech sounds by musical practice^14^. Subcortical responses to speech are more robust to noise in musicians than non-musicians, and this neural advantage correlates with better abilities to perceive speech in noisy background^15^. Overall, these studies suggest that the perceptual advantages induced by intensive music training rely on an enhancement of the neural coding of sounds, in both cortical and subcortical structures and extending to speech sounds.

Interestingly, musical experience has also been associated with better perception and production of sounds in foreign languages^16–18^. At the cortical level, the slight pitch variations of both musical (i.e. harmonic sounds) and non-native speech syllables (i.e. Mandarin tones) evoke larger mismatch negativity (MMN) responses in non-native musicians as compared to non-native nonmusicians^17,19^. At the subcortical level, Wong and colleagues (2007) have shown that American musicians have more faithful neural representation of the rapid variations of the pitch of Mandarin tone contours as compared to American non-musicians^20^. Moreover, this advantage correlates with the amount of musical experience.

Krishnan and colleagues’ work on the cross-domain effects between music and language experience revealed that long-term experience in pitch processing (e.g. in musicians and tonal language speakers) enhances the encoding of pitch regardless of the domain of experience^21,22^. These findings suggest that musical practice can compensate for the lack of language experience when it comes to processing foreign tonal speech sounds by sculpting the automatic neural coding of pitch through years of training. However, pitch is only one relevant acoustic dimension of speech, and musicians’ advantages in foreign language perception are not restricted to tonal languages^18,23,24^. This raises the question of whether musical experience compensates for the lack of language experience in non-tonal languages by improving the processing of other relevant speech features, such as formant cues that allow a listener to discriminate vowels.

To this aim, we compared subcortical electrophysiological responses to the English syllable [thae] between three groups of non-tonal language speakers: Americans non-musicians, French non-musicians, and French musicians. Since French speakers are familiar with English sounds, we chose a stimulus that is distant from the phonemic inventory of French, the English syllable [thae]—neither the consonant nor the vowel phonemes exist in French. In a previous study we compared frequency-following responses (FFR) to the syllable [thae] in American and French non-musicians^25^. Compared to French speakers, American non-musicians had more robust subcortical representations of the English phoneme [thae]. Thus, we hypothesized that American participants would confirm an advantage in neural encoding of linguistically-relevant features as compared to non-native speakers without musical practice, but that this language difference would be compensated by musical practice in non-native musicians. Specifically, we predicted that French musicians would resemble American non-musicians in their neural processing of [thae]. This should be visible in terms of differences in processing the harmonics for the consonant and of the formant harmonics for the vowel, thus allowing us to tackle the issue of selective versus global enhancement following musical training.

## Results

### Global spectral representation

**Figure 1:**
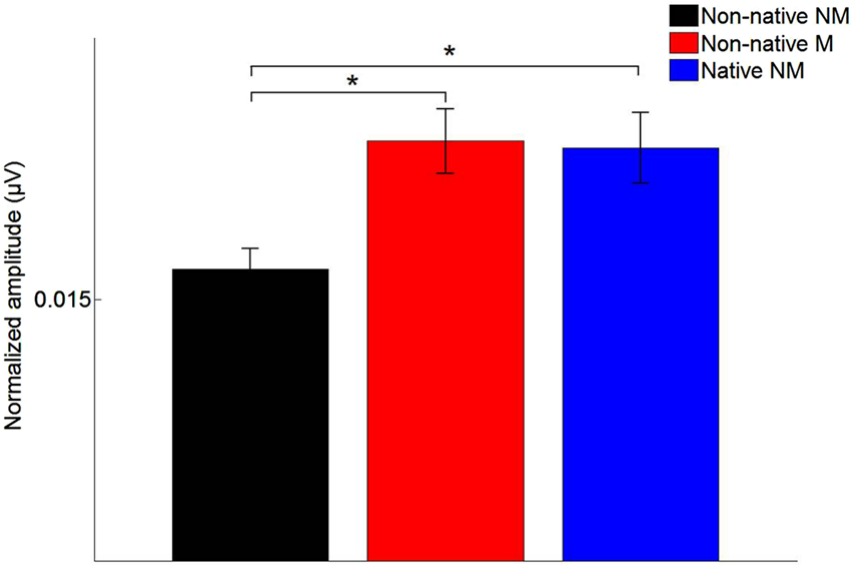
Global spectral subcortical representation of the English syllable [thae] averaged across consonant and vowel for different frequency bands (see Material and methods for more details). Non-native nonmusicians (black), non-native musicians (red) and native nonmusicians (blue). *p < 0.05; error bars represent ± 1 standard error.

Native nonmusicians and non-native musicians showed an overall stronger neural representation of the frequency components compared to non-native nonmusicians (*F*(2,37) = 3.40, *p* = 0.044, Fig. 1). More precisely, while non-native musicians and native speakers did not differ in terms of their subcortical representation of the spectral components (*p* = 0.9), they both showed a more robust representation compared to non-native nonmusicians (*p* = 0.032, *d* = 0.90 and *p* = 0.033, *d* = 0.92 respectively). There was also a significant main effect of time region and, more importantly, a significant interaction between time regions and frequencies (*F*(3,111) = 30.80, *p* < 0.0001), showing a different spectral pattern for each time region. The interaction terms were further analyzed using separate analyses for each time region.

### Fundamental frequency (F0)

**Figure 2:**
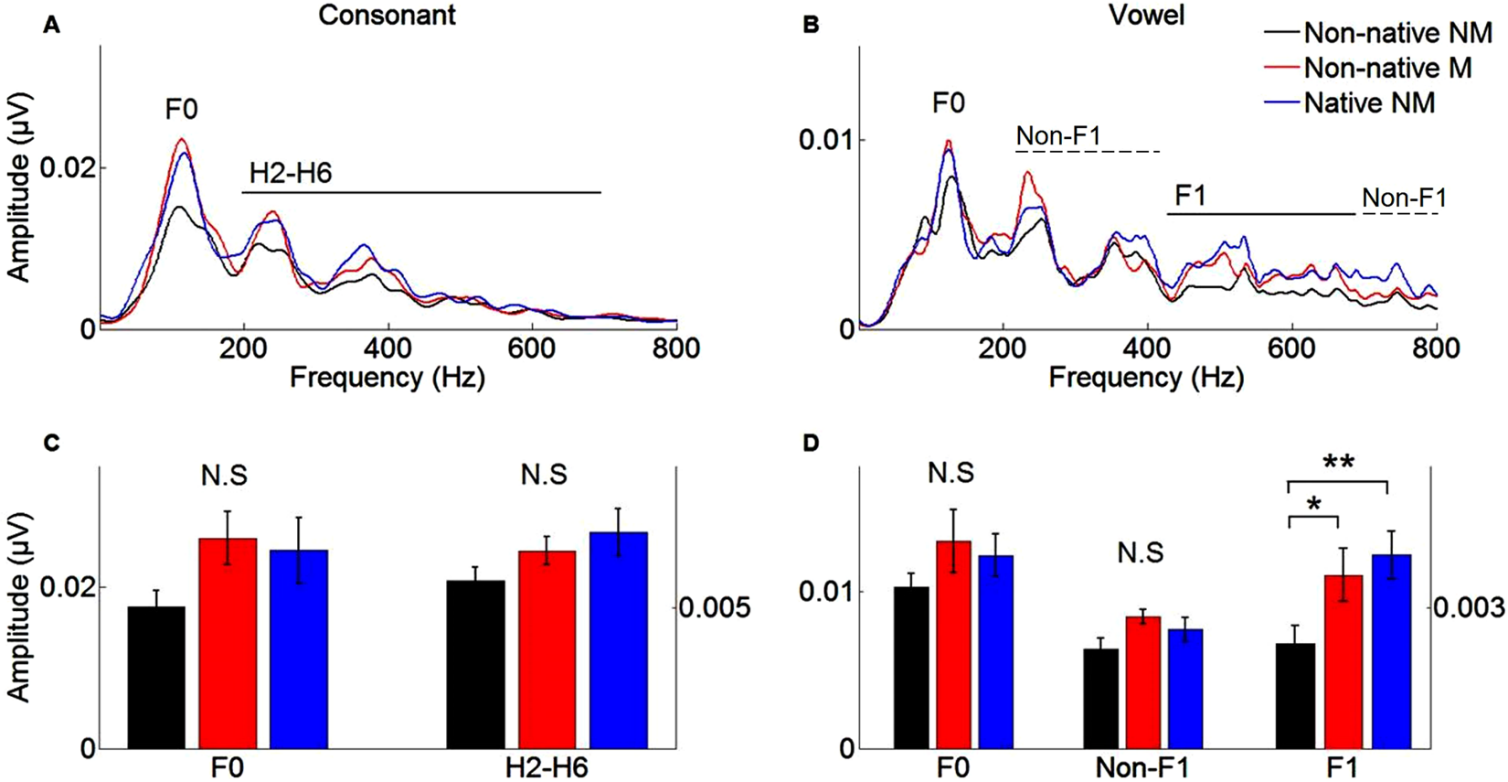
Spectral representations. **Top**: Fast Fourier transform of the neural response to the consonant (left panel) and the vowel (right panel) for non-native nonmusicians (black), non-native musicians (red) and native nonmusicians (blue). **Bottom**: Bar graphs corresponding to the fundamental frequency (F0) and its subsequent harmonics (H2-H6) for the consonant (left), and to the F0, the first formant (F1) and the non-formant frequencies (Non-F1) for the vowel (right). Left y axes correspond to the F0 and Non-F1 frequencies, right y axes correspond to the harmonics (H2-H6) and F1 frequencies. *p < 0.05; **p < 0.01; error bars represent ± 1 standard error.

The groups did not differ in F0 representation. Spectral analysis of the time region corresponding to the consonant and the vowel did not reveal any group difference in the encoding of the F0 (consonant: *F*(2,37) = 2.10, *p* = 0.137, Fig. 2A,C; vowel**:** *F*(2,37) = 1.19, *p* = 0.315, Fig. 2B,D).

### Consonant Harmonics (H2-H6)

The groups did not differ in harmonic representation. Analysis of time region corresponding to the consonant did not show any group difference on the spectral encoding of the harmonics (*F*(2,37) = 1.98, *p* = 0.152, Fig. 2A,C).

### Vowel Formant (F1) and non-formant (Non-F1) frequencies

Native nonmusicians and non-native musicians both had stronger representation of the formant than non-native nonmusicians. Indeed, analysis of time region corresponding to the vowel showed significant group differences in the spectral encoding of the formant frequencies (*F*(1,37) = 4.63, *p* = 0.016, Fig. 2B,D). While non-native nonmusicians exhibited poorer representation of the F1 compared to the native speakers and non-native musicians (*p* = 0.006, *d* = 0.90 and *p* = 0.042, *d* = 0.83, respectively), non-native musicians did not differ from native speakers in their neural representation of the F1 (*p* = 0.546). For the non-formant frequencies the main effect of group did not reach significance level (*F*(2,37) = 2.18, *p* = 0.127).

## Discussion

This study investigated the effect of both language and musical experience on the neural encoding of non-tonal speech sounds. The results demonstrate that (1) neural processing of formant frequencies is dependent on language experience; (2) most importantly, music experience partly compensates for a lack of language experience at an early (subcortical) stage of auditory processing.

In our previous study^25^, we have shown that language-dependent effects in subcortical structures are not restricted to pitch processing. French participants had more robust neural encoding of formant frequencies of their native language compared to American participants. Here we extend these findings by showing stronger neural representations of the first formant of an English syllable in American compared to French participants (nonmusicians). Importantly, spectral representations of formant frequencies (F1) that are relevant to the phonemic system are more strongly enhanced in native speakers whereas other non-relevant spectral features (non-F1 harmonics) are not affected by language experience. This is consistent with the hypothesis that language-dependent plasticity occurs as a function of what is functionally relevant to a listener. For instance, compared to English, Chinese speakers show stronger spectral encoding of the pitch of stimuli characterized by rapid changes that are lexically-relevant in Chinese Mandarin^21^. This specific neural enhancement has also been shown in musicians who are, for instance, particularly sensitive to the leading melodic voice in a chord^26^ or the pitch frequencies that correspond to a note along the diatonic musical scale^21^.

The major finding of this study is that, compared to native English speakers, non-native French musicians have similar subcortical representation of an American English syllable and this effect is mostly driven by the vowel formant frequency. In other words, musical practice seems to compensate for language-dependent effects at least at the early stages of neural processing. Previous work has mainly focused on transfer effects between musical experience and native language or tonal foreign language perception. For instance, musicians have more precise and distinct neural representations of native syllables that differ only on their second formant trajectories^24,27^, and this possibly allows for enhanced automatic processing of different consonant features in their native language^11^. Musical practice has also been associated with better perceptual abilities to categorize native vowels along a speech continuum, and these behavioral advantages coincided with more robust subcortical encoding of salient speech cues, such as voice pitch and formant frequencies^28^. Turning to foreign languages, several experiments examined the influence of musical experience on the processing of tonal languages^16^. These studies revealed that musicians have better perceptual abilities^29–31^, as well as more robust neural processing of non-native lexical tones^17,20^. While these results are not trivial, one may argue that the musicians’ advantage in processing lexical tones relates to a more accurate pitch perception and/or memory for pitch, because the F0 trajectories are both relevant in tonal languages and in music. However, to our knowledge, this is the first time the effects of musical practice on subcortical processing of non-tonal foreign languages has been investigated. The present results go beyond the previous literature on pitch processing by showing that musical experience strengthens the neural processing of non-tonal foreign languages along the specific phonetic dimensions that are linguistically-relevant (formants), as opposed to an overall gain in neural processing. Indeed, although the global effect is significant across frequencies (Fig. 1) and a trend is visible on Fig. 2D for F0 and non-formant frequencies, musical training only significantly affected formant frequency subcortical representations. While, based on previous literature^20^, one may expect differences to be visible on F0 representation, the lack of significant differences may also be due to the fact that the analysis of subtractive polarities maximizes the spectral response to the harmonics and formant frequencies at the expenses of lower frequencies such as the F0. Thus, while previous studies showed a global effect of musical experience on the F0, our study shows that these global effects are possibly accompanied by a selective enhancement of specific phonetic features.

These experience-dependent plasticity effects shed new light on the literature pointing to a possible effect of music training onto perception and production abilities in a second language. For instance, it has been shown that musicians outperform nonmusicians in discriminating foreign speech sounds and are also better in learning novel phonetic categories of that same foreign language^32^. A similar advantage has been shown with speech segmentation in both adults and children learning an artificial language^33^. Turning to speech production abilities, better neural sensitivity to acoustic differences in music materials and higher musical aptitude are associated with superior production in a second language^34,35^. Similarly, musically trained Indian children performed better on an English comprehension and vocabulary test than nonmusician children^36^. These studies demonstrate that musical experience positively affects multiple aspects of proficiency with a second language, such as phonological perception and production.

Music training may enhance the ability to process fine-grained, information-bearing spectral cues by facilitating precise and repeated engagement with sounds^2^. At least with respect to subcortical processing, this may compensate for a lack of language experience when processing non-native speech cues. Importantly, such enhanced processing is not a global enhancement of one or several acoustic features of a stimulus, but rather seems to be a selective enhancement of those features that are linguistically relevant.

Our results may also reflect top-down processes that heighten relevant and filter non-relevant incoming sensory information in subcortical structures. Anatomically, there are several projections from the cortex to subcortical structures that can support the top-down dynamics^37^. Krishnan and colleagues (2012) proposed a theoretical framework in which experience-dependent neural plasticity rely on both local mechanisms in the inferior colliculus^22^ (the presumed main neural generator of FFR^38^), and dynamic feedback and feedforward interactions between subcortical and cortical structures. Stimulus features that are behaviorally-relevant for the listener would activate local, feedforward, and feedback loops in a coordinated manner at each stage of processing. With growing experience, neural plasticity occurs in subcortical structures thus improving the neural processing of relevant acoustic cues.

In summary, we find that language-dependent effects occur along dimensions that are relevant to the listener, such as formant frequencies, and musical training can partly compensate for the lack of language experience by strengthening the neural subcortical processing of these linguistically-relevant cues bringing them to a level similar to a native speaker. One limitation of this study is that here we only tested two phonemes: the consonant [ð] and the vowel [æ]. These results should thus be extended to other speech sounds and to other languages. Nonetheless, these neural advantages in musicians are evocative of a rich literature documenting their better abilities in perception and production in a second language, and our results may provide a neural mechanism that underlies one or more of these advantages. These findings reinforce the link between music and speech, and support the hypothesis that music training benefits second-language acquisition and may thus play an important role in the educational system. Further work with a longitudinal approach rather than the cross-sectional approach used here should be carried on in order to understand to which extent music training modifies neural functions and to which extent pre-existing differences (such as musical aptitude) may also play a role.

## Material and Methods

### Participants

Forty-two (27 females and 15 males) young adults ranging in age from 18 to 29 years (mean age 22.5 ± 0.55 years), participated in the study. Fourteen (8 females and 6 males, mean age 21.8 ± 0.93 years) were native speakers of American English with no or limited amount of musical training (henceforth, native nonmusicians, mean years of practice: 2.9 ± 0.79 years). 18 were native French speakers (8 females and 10 males, mean age 23.3 ± 0.98 years) with no or limited amount of musical training (henceforth, non-native nonmusicians, mean years of practice: 2.2 ± 0.85 years) and 10 (6 females and 4 males, mean age 22.3 ± 0.79 years) were native French speakers musicians (henceforth, non-native musicians; mean years of practice: 13.4 ± 0.9 years; mean years of classical formal training: 10.9 ± 0.8). American participants were recruited at Northwestern University (Chicago, USA) and French participants were recruited at Aix-Marseille University (Marseille, France). French participants started learning English at school (mean age 10.83 ± 0.34 years). All participants were monolinguals and had no history of hearing, neurological, or psychiatric disorders. Inclusion criteria were a high-school level of education and click-evoked brainstem response latencies within lab-internal normal limits (5.41-5.97 ms; 100-μs click stimulus presented at 80 dB SPL). The three groups did not differ in term of click latencies (*p* = 0.28). The two French groups did not differ in term of the English language (school level, *p* = 0.57) nor in self-reported comprehension levels (*p* = 0.10). The non-musician participants were a subset of a pool of participants that participated in a previous study on the effects of native language on subcortical representation of speech^25^.

All methods were performed in accordance with the relevant guidelines and regulations. All experimental protocols were approved by the Local Ethics Committee (CPP Méditerranée Sud, A01490-49). Participants gave their informed consent and were paid for their participation.

### Stimulus

**Figure 3:**
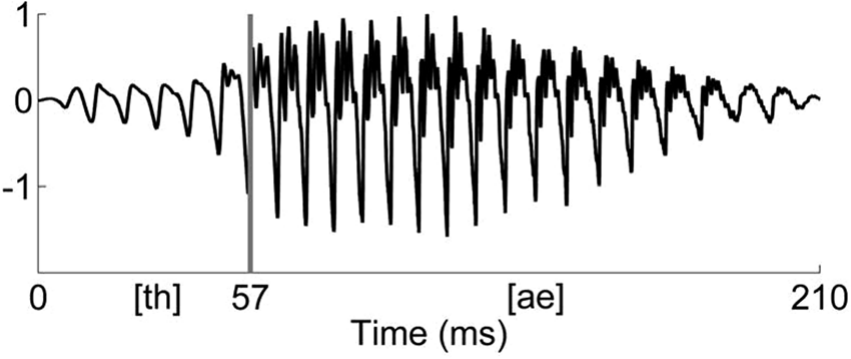
Waveform of the stimulus (normalized amplitude). The vertical gray line indicates the boundary between the consonant and vowel, as established according to the spectral changes by an experienced phonetician.

The stimulus used was the natural English syllable [ðæ] (consonant [ð] from “the” and vowel [æ] from “cat” with American pronunciation) recorded in an anechoic chamber by an American English male speaker (Fig. 3). This syllable was chosen because it is an « illegal » speech sound in French, which means that both the consonant and the vowel do not exist in French (i.e., [ð] and [æ] do not exist in French). This choice should maximize the differences between the two populations (French and American English) and should consequently maximize the expected effect of language experience on neural responses.

**Figure 4:**
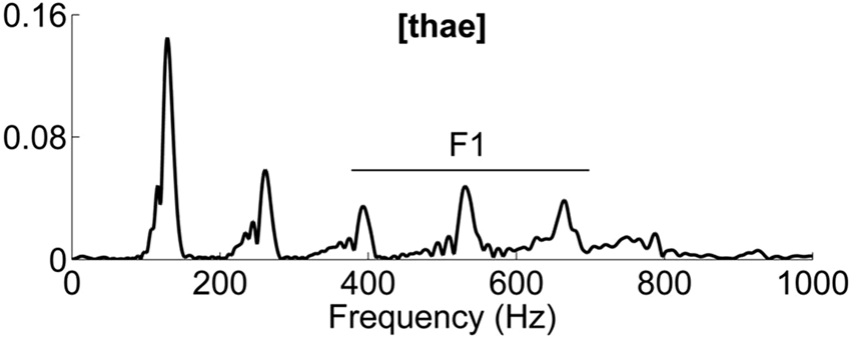
Fast Fourier transform computed on the whole stimulus (normalized amplitude). The horizontal line indicates the range of the first formant.

The total duration of the syllable was 209 ms. Frequencies of interest included the fundamental frequency (*F*0 at 129 Hz) and the second to sixth harmonics (*H*2-*H*6 at 261, 393, 531, 665, 787 Hz respectively), encompassing the first formant range (*F*1 ranging from 400 to 700 Hz) important for phoneme discrimination in non-tonal languages (see FFT Fig. 4). The second formant range exceeded the phase-locking limit of the auditory brainstem and was thus not taken into consideration in further analyses^39,40^.

### Electrophysiological recordings

The stimulus was presented monaurally to the right ear at 80 dB SPL at a rate of 3.3 Hz through magnetically shielded insert earphones (ER-3A, Etymotic Research) while participants sat in a comfortable reclining chair in an electrically-shielded, sound-attenuated room. Participants were instructed to watch a subtitled movie of their choice to maintain relaxation and prevent drowsiness. Brain responses were collected at 30 kHz sampling rate using Microvitae recording system (µV-ABR) with three Ag-AgCl scalp electrodes in a vertical montage (Cz active, forehead ground, and right earlobe reference). Electrode impedances were kept <5 kΩ. A total of six-thousand sweeps were collected (three thousands for each stimulus polarity). The experiment, including electrodes placement, lasted approximately 90 minutes for each participant. The stimulus polarities were alternated across trials in order to minimize the contribution of stimulus artifact and cochlear microphonic^39,41^. One of the authors (BI) was in charge of data acquisition in both countries using the same portable EEG system. This prevents the possibility of having a bias due to different experimental setups, participant preparation, and instructions.

### Data analysis

All analyses were performed using custom MATLAB scripts (MATLAB R2013b, MathWorks Inc). First, electrophysiological recordings were bandpass filtered from 70 to 2000 Hz (12 dB/octave roll-off) using a Butterworth filter. Then, sweeps with activity exceeding ± 30 µV were rejected as artifacts and the responses were baseline-corrected to the pre-stimulus period (−30 to 0 ms). Neural responses were then averaged over a −30 to 230 ms window.

Power spectral density was computed via Fast Fourier transform on the individual averages obtained by subtracting the responses to the two polarities. This choice, different from the one that we previously adopted^25^ was done in order to maximize the spectral response to the harmonics and formant frequencies^41,42^ that are the most important features for consonant and vowel encoding. FFTs were performed on two independent time regions of the response (consonant and vowel) because previous studies have shown that these two time regions are affected differently by musical and language experience^25,43^. These time regions were defined on the basis of the stimulus by a phonetician also taking into account a 10 ms neural delay in the response: consonant (20–67 ms) and vowel (67–220 ms).

For each time region, the maximum spectral amplitudes of the fundamental frequency (F0) and its second to sixth harmonics (H2–H6) were extracted in a bandwidth of 20 Hz surrounding the peak in the stimulus fast Fourier transform (e.g. for a peak at 118 Hz, values were extracted between 108 and 128 Hz)^43^. For the consonant, the five values extracted from *H*2 to *H*6 were then averaged to form a global measure of harmonics’ representation. For the vowel, harmonics falling within *F*1 range ± 20 Hz were averaged to form a global measure of *F*1 spectral representation (*H*3-*H*5), while harmonics falling outside *F*1 range were averaged to form a global measure of non-formant spectral representation (*H*2, *H*6). Testing the representation of different acoustic features was important in order to determine whether the effect of language and musical experience would be specific to phonetic dimensions that are linguistically-relevant (formants), or whether it would rather be an overall gain in neural processing visible on non linguistically-relevant features.

### Statistical analyses

The analyses of variance (ANOVAs) were all performed using group as a between participants factor (non-native nonmusicians vs. non-native musicians vs. native nonmusicians) and two time regions (consonant and vowel) and spectral amplitude of frequencies of interest as dependent variables (*F*0, *F*1, non-formant harmonics and mean of harmonics). First, we ran a complete model to investigate the global effect of language and musical experience, including group (3 levels) as between participants factor, and time regions (2 levels) and spectral components (4 levels) as within participants factors. Then, in order to gather a more precise insight of the effect of group we ran two separate ANOVAs for the consonant (group, 3 levels and spectral components, F0 and mean of harmonics, 2 levels) and vowel (group, 3 levels and spectral components, F0, F1 and non-formant harmonics, 3 levels). *Post-hoc* tests were used when appropriate (Fisher LSD).
